# Counteracting uncertainty: exploring the impact of anxiety on updating predictions about environmental states

**DOI:** 10.1007/s00422-025-01006-4

**Published:** 2025-02-20

**Authors:** David Harris, Tom Arthur, Mark Wilson, Ben Le Gallais, Thomas Parsons, Ally Dill, Sam Vine

**Affiliations:** School of Public Health and Sport Sciences, Medical School, University of Exeter, St Luke’s Campus, Exeter, EX1 2LU UK

**Keywords:** Gaze, Stress, Eye tracking, Bayesian, Predictive Processing

## Abstract

Anxious emotional states disrupt decision-making and control of dexterous motor actions. Computational work has shown that anxiety-induced uncertainty alters the rate at which we learn about the environment, but the subsequent impact on the predictive beliefs that drive action control remains to be understood. In the present work we tested whether anxiety alters predictive (oculo)motor control mechanisms. Thirty participants completed an experimental task that consisted of manual interception of a projectile performed in virtual reality. Participants were subjected to conditions designed to induce states of high or low anxiety using performance incentives and social-evaluative pressure. We measured subsequent effects on physiological arousal, self-reported state anxiety, and eye movements. Under high pressure conditions we observed visual sampling of the task environment characterised by higher variability and entropy of position prior to release of the projectile, consistent with an active attempt to reduce uncertainty. Computational modelling of predictive beliefs, using gaze data as inputs to a partially observable Markov decision process model, indicated that trial-to-trial updating of predictive beliefs was reduced during anxiety, suggesting that updates to priors were constrained. Additionally, state anxiety was related to a less deterministic mapping of beliefs to actions. These results support the idea that organisms may attempt to counter anxiety-related uncertainty by moving towards more familiar and certain sensorimotor patterns.

## Introduction

Both dextrous motor actions and control of our visual system are thought to depend on predictions about future states of the world and our own body (Wolpert and Flanagan 2001; Shadmehr et al. 2010; Adams et al. 2013). Skilled movement also depends on the ability to flexibly *adapt* and *update* those predictions according to new contexts or new sensory evidence. As anxious emotional states can bias the way in which we make and update predictions (Cornwell et al. 2017; Hein et al. 2021; Hein and Herrojo Ruiz 2022), they can disrupt motor actions with potentially damaging consequences (Harris et al. 2023b). It is well known that during highly pressurised situations, such as a job interview or the final of a sporting event, people can experience drastic breakdowns in task performance (Beilock and Carr 2001; Nieuwenhuys and Oudejans 2012; Payne et al. 2018). In the present work we sought to understand the impact of anxiety on predictive (oculo)motor control mechanisms. We first outline theoretical approaches that have described anxiety in terms of uncertainty or entropy and how sensorimotor control might reflect an active attempt to resolve that uncertainty.

Anxiety is a negative emotional response to a perceived threat (Eysenck 2013; Grupe and Nitschke 2013). It is often characterised along cognitive (worry) and somatic (physiological arousal) dimensions. Anxiety can lead to distinct difficulties learning about the world and making decisions (Bishop 2007; Carleton 2016), and can particularly disrupt the ability to adapt to changing task conditions, especially in individuals with an intolerance of uncertainty (Browning et al. 2015; Huang et al. 2017; Hein et al. 2021). One potential explanation for these effects is that anxiety may alter the way in which we make predictions about the world around us. For instance, several theoretical accounts have characterised anxiety as closely related to situational estimates of uncertainty and a (perceived) inability to reliably predict the absence of threats (Grupe and Nitschke 2013; de Berker et al. 2016; Cornwell et al. 2017; Seriès 2019; Lawson et al. 2021). For instance, Hirsh et al.’s. (2012) *Entropy Model of Uncertainty* (EMU) describes uncontrollable or unpredictable situations as creating an aversive high-entropy state, in which an organism experiences a reduced ability to predict successive states (e.g., sensory outcomes) based on the current state. According to the EMU, organisms find such uncertainty aversive and experience it subjectively as anxiety. Research in this vein therefore conceptualises anxiety as an *epistemic* emotion, that is arising from our engagement with knowledge, learning, or the need to reduce uncertainty (Miceli and Castelfranchi 2005).

The EMU characterisation of anxiety aligns closely with other dynamical systems approaches like the *Free Energy Principle* (FEP) and *Active Inference*, which similarly conceptualise the goal of a cognitive-behavioural system as the minimization of internal entropy (or ‘free energy’) and an increase in prediction success (Friston 2009; Friston et al. 2010; Clark 2013). From this perspective, to cope with instability (e.g., during anxiety) organisms should seek to return to familiar low-entropy states. Under the FEP (Friston 2010), this tendency to return to low entropy states is, by definition, true of a self-organising system as they must resist dissipative forces (i.e., states of high entropy) to maintain their own integrity and existence. One way to achieve this goal in the face of anxiety-induced uncertainty, is to adopt behaviours like withdrawal or avoidance to evade the aversive stimulus. Alternatively, the organism may seek out new perceptual information that will disambiguate any uncertainty (e.g., looking under the bed to check for monsters). These uncertainty-reducing behaviours can sometimes be detrimental, such as when a sportsperson’s movements become rigid and constrained (Harris et al. 2023b), but they may also be beneficial, enabling faster belief updating (e.g., Behrens et al. 2007)), or neutral, as in cases of task-irrelevant uncertainty reduction (e.g., Miceli and Castelfranchi 2005).

It has previously been suggested that an adaptive response in times of uncertainty is to modify the rate with which you update your beliefs about the world; learning faster allows one’s model of the world to better reflect the current ‘reality’ of an environment (Behrens et al. 2007). Neurocomputational work has illustrated that this is indeed the case for perceptual learning (Lawson et al. 2021) where learning rates (i.e., the speed at which we revise prior expectations) appear to be increased by noradrenergic up-regulation of prediction error signal strength. What remains to be determined, is whether anxiety-related uncertainty similarly induces faster updating of predictive aspects of sensori*motor* control,^1^ or if these might instead be impaired. While quickly revising predictions may be beneficial in some instances (e.g., when conditions are changing or inherently unstable), a constantly high learning rate would generally disrupt the formation of appropriate action beliefs or models (i.e., by limiting a person’s ability to disregard atypical or uncharacteristic task outcomes). As a result, participants may display highly variable and reflexive sensorimotor responses, which correspond less accurately with the surrounding performance context. Moreover, it has previously been reported that participants will *constrain* movement degrees of freedom when placed in more unpredictable task environments as an active attempt to reduce uncertainty (O’Sullivan et al. 2009; Arthur and Harris 2021). These findings align with theoretical models of impedance control in the motor system where co-activation of antagonistic muscle pairs is a natural response to unpredictable perturbations (Mitrovic et al. 2010). There is initial evidence that updating of predictions that guide sensorimotor control may indeed be slowed. Using a combination of EEG and computational modelling, Sporn et al. (2020) demonstrated that rates of motor learning were *reduced* under anxiety, and that anxious states impaired reward processing and the updating of predictions about rewards associated with motor sequences. Similarly, Ellmers et al. (2024) found that participants’ approach velocity in the moving escalator paradigm (Reynolds and Bronstein 2003) was updated more slowly under state anxiety. In essence, constraining updates to sensorimotor predictions could help the organism stay in familiar states when uncertainty is already high. Consequently, there may be important differences in how perceived uncertainty influences prediction updating in overt motor and oculomotor actions compared to simpler (perceptual) associative learning tasks.

### The present study

**Fig. 1:**
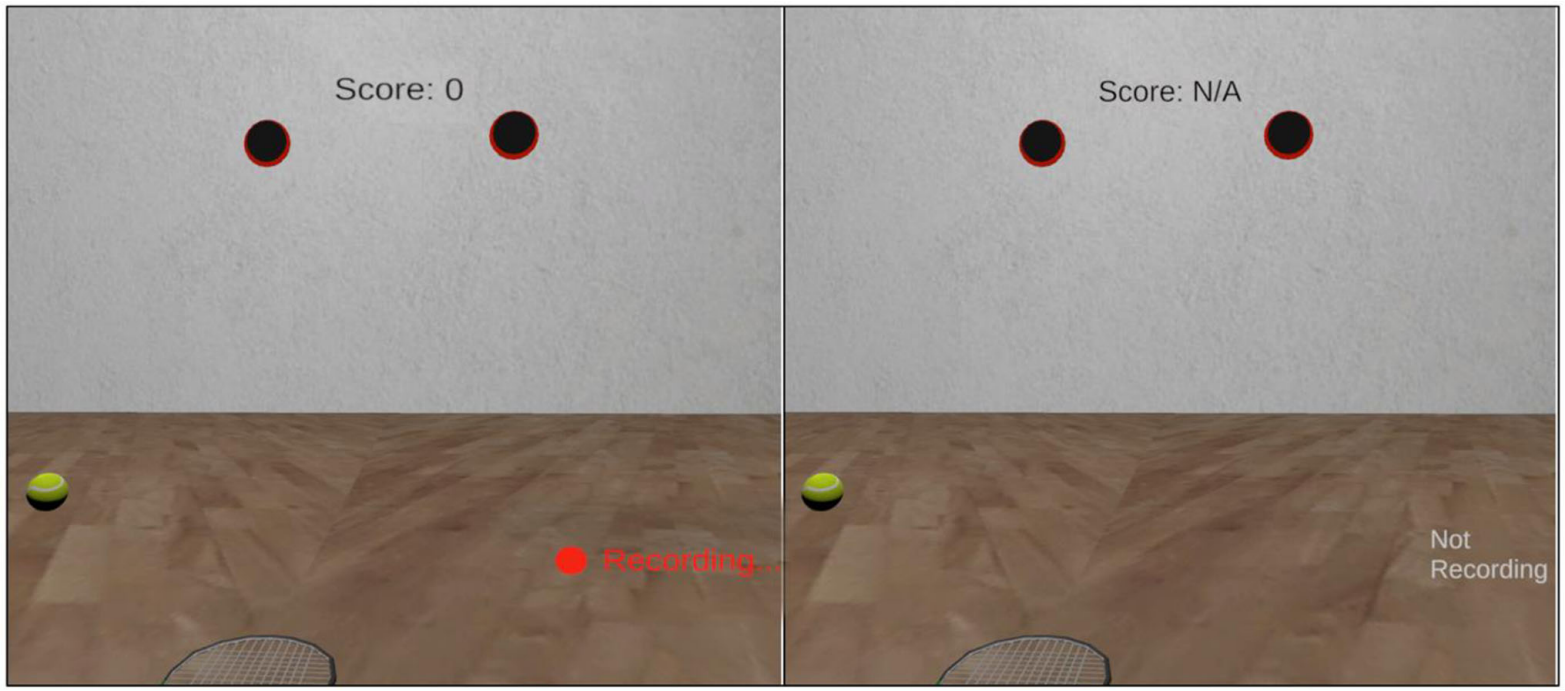
Virtual reality task environment for the high (left) and low (right) pressure conditions. Note: The ball was projected from one of the two locations on the front wall. The ball passed the player without bouncing and they were instructed to intercept it with the racquet (videos of hit and miss trials of the task are available online: https://osf.io/2amdv/). The score counter was displayed right at the top of the participant’s field of view and increased with every successful interception during the high pressure condition. In the low pressure condition (right) the score was not counted, and the red ‘recording’ text was replaced with ‘not recording’

Previous studies have shown that visual attention can be disrupted during anxious states, leading to subsequent breakdowns in task performance (Wilson et al. 2009; Laretzaki et al. 2011; Allsop and Gray 2014). An open question is what effect anxious states have specifically on the predictive mechanisms that underpin gaze control (Friston et al. 2012; Henderson 2017). This was the focus of the present study, in which participants performed an interceptive task in a virtual reality environment (see Fig. 1) while we manipulated performance incentives to induce situational pressure (as in Wood and Wilson 2010; Allsop and Gray 2014). Our focus was therefore on performance anxiety as an epistemic emotion (Miceli and Castelfranchi 2005), arising from uncertainty about the ability to execute the task and the associated monetary and social evaluative consequences. We measured subsequent effects of performance pressure on physiological arousal, self-reported state anxiety, and eye movements. To examine changes to predictive beliefs underpinning gaze control, we adopted computational modelling approaches that are increasingly being employed in psychiatry and clinical neuropsychology (Henco et al. 2020; Smith et al. 2020a; Parr et al. 2021) as a method for estimating how expectations change over time.

Our hypotheses were based on EMU and FEP characterisations of anxiety as a state of entropy which organisms will seek to resolve (Hirsh et al. 2012; McGovern et al. 2022). Our primary hypothesis was that updates to predictive eye movements (relating to the upcoming release location of a projectile) over trials would be impaired during state anxiety, as previous findings have indicated that participants may constrain both their movements and their rate of prediction updating under uncertainty (Sporn et al. 2020; Arthur and Harris 2021). We also examined some secondary eye movement metrics (before and after the release of the projectile) to understand how anxiety influenced oculomotor control. If participants are indeed uncertain about their ability to predict future states during anxiety, we would also expect impaired anticipatory tracking of predictable moving targets during the interceptive task. Given the importance of prior expectations in interceptive motor tasks (e.g., see Diaz et al. 2013), such uncertainty would likely be reflected in more variable, stimulus-driven (as opposed to prediction-driven) scan patterns under anxiety. We therefore hypothesised that high pressure conditions would also lead to greater variance and higher entropy of eye movements *prior* to the release of the interception target.

## Methods

### Design

We used a repeated measures design in which all participants took part in both high and low pressure conditions, the order of which was counterbalanced across participants.

### Transparency and openness

We report sample size determination, data exclusions, and all manipulations in the study. All data and analysis code are available at: https://osf.io/2amdv/. The hypotheses were not preregistered. Data were analysed using RStudio v1.4.1106 (R Core Team 2017).

### Participants

Thirty participants (12 female; M_age_ = 20.8 years; SD_age_ = 1.2; range = 18–23 years) took part in the study. Participants were recruited from the undergraduate student population at the host University. Eye tracking data was lost for one participant in one condition due to a recording error. In a previous study, we observed effects in the range of *R*^2^ = 0.03-0.06 for changes in predictive gaze position in this task (Harris et al. 2023a). Power curves using the simr package for R (Green and MacLeod 2016) showed that for linear mixed effects models (with participant as a random factor), 36 trials for 30 participants was sufficient to detect effects at the lower end of this range with more than 85% power (see power curves in the supplementary files: https://osf.io/2amdv/). Written informed consent from all participants was obtained in accordance with British Psychological Society guidelines, and the study received approval from the Departmental Ethics Committee (University of Exeter, UK; REF: 520,338). The study methods closely adhered to the approved procedures and the Declaration of Helsinki. Data collection was completed between December 2022 and May 2023.

### Task and materials

The experimental task consisted of manual interception of a projectile performed in virtual reality (VR). The task was a simplified racquetball game in which participants were instructed to intercept a ball projected from one of two possible locations at the front of the court using a virtual racquet operated by a tracked hand controller. Participants were given no prior cues as to which of the two locations the ball would appear from on each trial, but there was an auditory tone signalling the start of each trial. Overall, there was a 50/50 split of left and right sided trials which were presented to the participants in one of four pseudo-randomised orders (counterbalanced across participants). However, the trial orders were designed to be volatile, with periods where the projection location was clearly biased to one side or the other to induce probabilistic learning. Illustrations of these orders are available in the online supplementary files (https://osf.io/pwmhy). Participants were given no information about the statistical structure of the task or the optimal strategy, so that any emerging eye movement patterns reflected a spontaneous response to the task structure. Balls were 5.7 cm in diameter and resembled the visual appearance of a real-world tennis ball. These balls remained visible from the moment of their release, through to the end of each trial, meaning that participants could observe the true projected location regardless of whether they intercepted or missed the target stimulus. The visible racquet in VR was 0.6 × 0.3 × 0.01 m, although its physical thickness was exaggerated by 20 cm to facilitate the detection of ball-to-racquet collisions.

The virtual environment, which had the visual appearance of an indoor racquetball court, was developed using the gaming engine Unity (v2019.3.1f; Unity Technologies, San Francisco, CA) (see Fig. 1). The same task has been used to study predictive eye movements in previous papers (Harris et al. 2022, 2023a). The task was displayed using an HTC Vive head-mounted display (HTC Inc., Taoyuan City, Taiwan). The HTC-Vive is a high-precision, consumer-grade VR system which has proven valid for small-area movement research tasks (field of view: 110°, accuracy: 1.5 cm, jitter: 0.5 mm, latency: 22 ms; (Niehorster et al. 2017; Schuetz and Fiehler 2022). Two ‘lighthouse’ base stations projecting infra-red light act as a reference point to record movements of the headset and hand controller at 90 Hz. The headset features inbuilt eye-tracking, which uses binocular dark pupil tracking to monitor gaze at 120 Hz (spatial accuracy: 0.5–1.1°; latency: 10 ms, headset display resolution: 1440 × 1600 pixels per eye). Gaze was calibrated over five virtual locations prior to each condition, and upon any obvious displacement of the headset during trials.

## Measures

### Anxiety

***State anxiety.*** State anxiety (S-ANX) during each experimental condition was assessed using The Anxiety Thermometer (Houtman and Bakker 1989), which requires participants to report their anxiety at the current moment on a simple scale ranging from 0–10 (0 = not at all anxious; 10 = extremely anxious). This is a time-efficient measurement tool that has been used in related studies that presented low- and high-threat conditions (Wood and Wilson 2010).

***Heart rate.*** Heart rate was used to index sympathetic nervous system activation changes related to anxious states. Participants wore a Polar H10 Heart Rate Sensor (Polar Electro Oy, Kempele, Finland) around the chest, with data recorded via a Polar 2 Heart Rate Monitor Transmitter Watch (Polar Electro Oy, Kempele, Finland). The Polar H10 has a 100 Hz sample rate and values were recorded from the watch at 1 min intervals and averaged across each block of trials. Reported values were baselined to participants’ resting HR, which was taken over a 2 min period while they were seated.

### Eye tracking

Gaze-in-world coordinates (i.e., the intersection point of the gaze vector with the task environment) were recorded from the VR eye tracking system and then denoised with a three frame moving median filter and a second-order 15 Hz lowpass Butterworth filter (Fooken and Spering 2020). We subsequently calculated the following metrics.

#### Predictive eye movements

The primary variable of interest, was the predictive eye movement that took place immediately before ball release. *Predictive gaze location*, was defined as the horizontal (x-coordinate) gaze position (i.e., gaze vector intersection point with the world) at the termination of the auditory tones, averaged over a 50 ms window. Following Harris et al. (2023a) this was taken as the most critical moment for anticipating the projection location and therefore the critical eye movement feature that would reveal participants’ prediction about the upcoming trial. The predictive gaze location variable was then used as an input to model the updating of sensorimotor predictions over trials (described in the *Active Inference Modelling* section below). Our previous work using this task has shown that participants either make an overt prediction with their eyes (directing gaze to one of the two projection locations) or make no prediction and ‘hedge their bets’ by looking directly between the two locations (Arthur et al. 2024). The fact that participants do not *have to* look at either location means that trials in which they choose to direct gaze towards one of the two locations is a strong indication of their expectation about the most likely upcoming release location. We therefore treated predictive gaze location as a discrete variable (left/right/centre); the central half of the space between the two locations (10.9° of visual angle) was treated as non-predictive and those outside as left or right predictions (as in Arthur et al. 2024). This approach allowed us to adopt the widely used discrete state space approach to modelling active inference (Smith et al. 2020b, a) to infer the underlying belief (most likely location) from the eye position (anticipating left/right/neither).

#### Pre-release variability and complexity

**Fig. 2:**
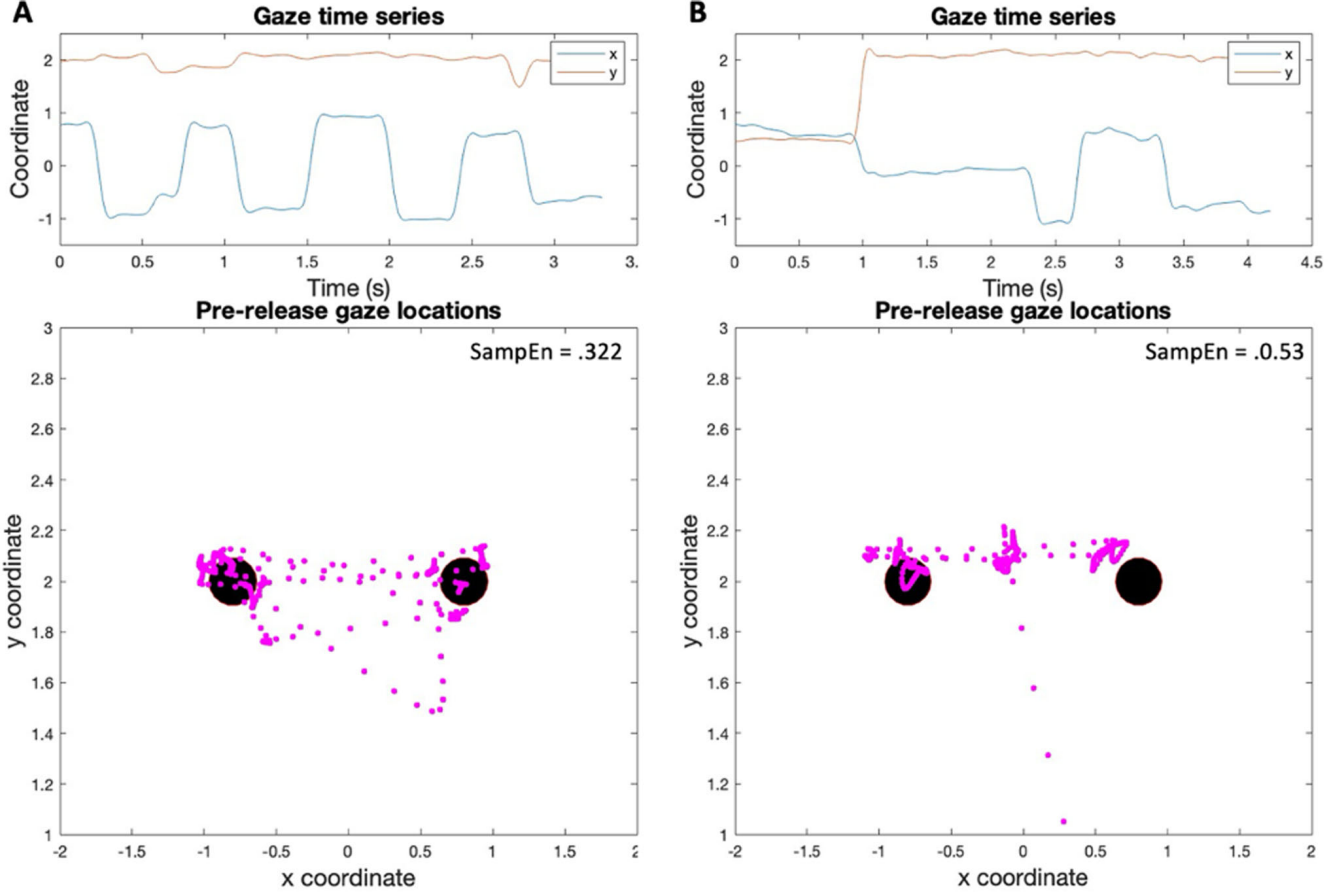
Pre-release gaze position from a high entropy (left) and low entropy (right) trial for participant 1. Note: The top figures show time series plots of the x and y coordinates of gaze position prior to ball release for two selected trials. Panel A (left) illustrates a high entropy trial where gaze switched quickly between the two locations and the participant searched the task space more randomly. Panel B (right) illustrates a low entropy trial where eye position was more stable and there were fewer changes of location. The lower plots show the corresponding gaze traces and entropy values

To address our secondary hypotheses, we examined patterns of eye movement shifts prior to release relating to (i) variability of position and (ii) complexity (entropy) of the signal (see example plots in Fig. 2). Variability of eye position has been used in previous studies (e.g., in driving tasks; Wilson et al. 2008) to indicate anxiety-induced disruptions to gaze. *Gaze variability* was defined as the 2-dimensional standard deviation of the gaze intersection position for the period from the trial start until the ball release cue. Here, we were interested in the eye movements that occurred just *before* the ball was projected from one of the two wall locations, as they would indicate participant’s state predictions (e.g., about where the ball would emerge from) and expected uncertainty (i.e., relating to the environment and its ‘hidden’ task probabilities). *Gaze entropy* was calculated to index signal complexity, for which we adopted a sample entropy measure calculated from the natural logarithm of the conditional probability that a series similar for *n* points remains similar at the next point (see (Richman and Moorman 2000). Entropy was calculated separately for horizontal and vertical planes. Entropy can be used to index the level of randomness or complexity in biological time series data, including eye tracking, and has previously been used to detect changes in visual scanning during anxious states (Allsop and Gray 2014; Vine et al. 2015). Sample entropy is generally robust to variations in sample size so recording durations do not need to be exactly matched. Higher entropy values (in bits) would suggest a more complex signal, indicative of more random and reflexive (i.e., less predictable) movements over time.

#### In-flight tracking (pursuit gain)

For high speed targets (such as the ball in this task) close tracking of the target’s in-flight trajectory may only be achieved through anticipation of its future location (Fooken et al. 2021). Here, anticipatory head and eye movements can facilitate continuous smooth pursuit dynamics, while preventing a person’s point of gaze from significantly ‘lagging behind’ the target. To index whether eye position was keeping up with the target in this way, we calculated a measure of *pursuit gain*. Following Palidis et al. (2017), this was computed as the saccade-free eye velocity divided by target velocity (i.e., values of 1 indicate that the eye perfectly kept pace with the ball). To do this, periods during which a saccade were made (identified as portions of data where gaze acceleration exceeded five times its median absolute acceleration; Mann et al. 2019; Arthur et al. 2021) were firstly removed from the data. Following Fooken and Spering (2019), gain values were then calculated for the closed-loop portion of tracking (i.e., based on observed motion feedback; (Palidis et al. 2017) which was defined as 140 ms after ball release.

#### Active inference modelling

To probe the mechanisms behind the trial-to-trial updating of predictions we fit a computational model to the predictive gaze location variable. The purpose of this model was to use the observed behaviour (eye position) to infer an unobserved variable (belief about the next release location). We therefore chose a gaze variable (position before release) that had a clear mapping to a task-relevant belief (release location) (as in Harris et al. 2023a). A Bayesian generative model of perception derived from the Markov decision process (MDP) formulation of active inference (Da Costa et al. 2020) was chosen to align with the theoretical approach of the paper. This approach has previously been used to model individual characteristics of active inference in clinical states of depression and anxiety (Smith et al. 2020b, a; Taylor et al. 2023). It has also been used for predictive eye movements in a task similar to the one reported here (Arthur and Harris 2021).

Active inference provides a principled way of modelling behaviour as the minimisation of free energy, combining Bayesian inference and information-theoretic principles. However, it is worth highlighting that it is not the only computational framework leveraging entropy-based measures. For instance, reinforcement learning has been adapted to recast reward maximisation as control as inference (CAI), leveraging variational inference to model how an agent infers optimal action trajectories (Tschantz et al. 2020; Millidge et al. 2020). These approaches have many similarities to active inference but differ in the understanding of ‘reward’; active inference approaches incorporate ‘rewards’ into the generative model as prior preferences and seek to minimise their divergence from expected observations, instead of optimising a value function over external rewards. Here, we focus on active inference due to its unique ability to model anxiety-related processes as arising from the interplay of uncertainty reduction and epistemic value (e.g., balancing exploration and exploitation), making it particularly well-suited for examining performance anxiety.

**Fig. 3:**
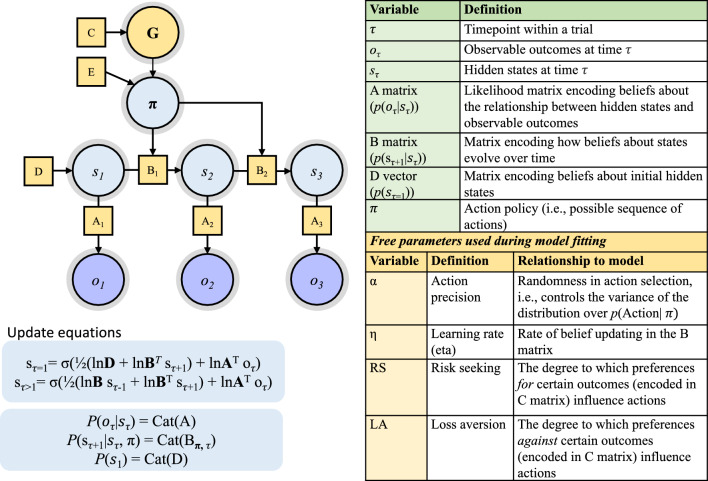
Directed acyclic graph illustrating the generative structure for the partially observable Markov decision process model. *Notes*: The model outlines inferences about projection locations based on active inference behaviours (eye position). Following the notation of Smith et al. (2021), circular nodes correspond to state, observation, and action policy variables, with arrows indicating dependencies between variables. Squares indicates factors mediating conditional relationships and take the form of matrices in the model. Observation and state subscripts correspond to time points in a trial (τ). When τ > 1, the B matrix from τ − 1 acts as an empirical prior, playing the same role as the D vector at τ = 1. Action policy selection (π) depends on the expected free energy associated with each policy (G), which in turn depends on prior preferences for outcomes (C), as well as expected precision of action policies (E). Policies expected to i) minimize uncertainty over states and ii) maximize the probability of preferred observations will have the highest probability of being selected. Sigma (σ) in the update equations (bottom left) refers to a SoftMax function (normalized exponential). More extensive descriptions of the model parameters in the table are provided in the supplementary files: https://osf.io/2amdv/

Such computational modelling of behavioural data allows us to estimate the processes that generated that data and therefore move towards a better understanding of underpinning mechanisms. This modelling approach requires specifying a generative structure for the inference process of interest (see Fig. 3), inputting observed values for known parameters, and optimising other, unobserved, model parameters to best explain the data. In the current case, the predictive eye movements that participants made (left/right/centre), and their subsequent observations (left or right outcome) are known. Given these known values, a gradient descent optimisation routine can be used to estimate participants’ ongoing beliefs about the most likely release location, the best courses of action available to them, and the rate at which these beliefs were updated over time.

In the model (see Fig. 3) beliefs about states of the world (s) are estimated over timepoints *within* a trial (τ) and across trials (*t*). The schematic in Fig. 3 represents a single trial, in which prior expectations about the release locations(*p*(S_1_); D) are encoded as a categorical distribution over possible states of the world (i.e., release location). Transitions between states (*p*(s_*τ*+1_| s_*τ*,_π); B) are updated over trials based on the likelihood matrix ((*p*(o_*τ*_|s_*τ*_)); A), which maps the probability of hidden states given observed instances. The partially-observable MDP model takes a set of initial parameters specified by the experimenter – e.g., prior expectations about release location (see online supplementary files for starting priors: https://osf.io/2amdv/) – then iteratively updates beliefs (according to Bayes rule) following the known actions and outcomes.

Each trial was modelled as consisting of three timesteps: τ = 1 was the start state before any observations had been made; τ = 2 was the cue timepoint when participants hear the onset cue and where the eye position is taken to be a prediction about release location; and τ = 3 is the ball appearance timestep when participants observe which release location was correct. Models did not extend past the observation of the ball appearance (i.e., into the swing of the racquet) because task related uncertainty effectively ended with the ball observation. At τ = 3, the participant observed the true release location and inferred whether the true state was left or right. That is, they inferred a posterior distribution over *P*(*s*_*τ* = 3_) that assigned a probability to the left versus right state, based on a Bayesian integration of prior beliefs about transitioning to the respective states *P*(*s*_*τ*_ _= 2_|*s*_*τ*_ _= 1_) and *P*(*o*_*τ*_|*s*_*τ*_) (i.e., the likelihood mapping). The inferred posterior formed the prior for the next trial. Bayesian belief updating in the model was based on the following equations for the timepoints (τ = 1 and τ = 2–3, respectively):

$$ \overline{\user2{s}}_{\tau = 1} = {\varvec{\sigma}}\left( {\frac{1}{2}\left( {\ln D + \ln B \cdot s_{\tau + 1} ) + {\text{ln }}A \cdot o_{\tau } } \right.} \right) $$

$$ \overline{\user2{s}}_{\tau = 2 - 3} = {\varvec{\sigma}}\left( {\ln B s_{\tau - 1} + {\text{ln }}A \cdot o_{\tau } } \right) $$

Here, ${\varvec{\sigma}}$ indicates a SoftMax function which converts the belief to a proper probability distribution and *ln* refers to the natural logarithm. Posteriors over states were estimated using an optimization routine (gradient descent) that minimises free energy in the model. Additional technical details are described in Fig. 3. By fitting the generative model to real participant data, we were able to identify a set of parameters that enabled model predictions to best match observed behaviour (i.e., maximising *p*(*participant be*ℎ*avior*|*model*)). For further details on the mathematical formalism of these models, see Friston et al. (2017), Smith et al. (2020a), or an introductory review by Smith and colleagues (Smith et al. 2022).

After identifying the model structure, we compared different combinations of free parameters to determine the best approach for fitting to participant data. We examined five possible free parameters and compared model fits with different combinations of these values. Full details of the competing models and the comparisons are available in the supplementary files (https://osf.io/2amdv/ – supplementary 5). Successive models were compared using a Bayesian random effects model (spm_BMS.m function: Statistical Parametric Mapping 12 toolbox; Wellcome Trust Centre for Neuroimaging, London, UK, http://www.fil.ion.ucl.ac.uk/), which assesses the variational free energy of each model fit and returns the relative probabilities (e.g., [0.9, 0.1]) of the better fit (the protected exceedance probability; Rigoux et al. 2014). The winning model had three free parameters: η (learning rate), α (action precision), and loss aversion. The parameter η describes the magnitude of belief updating at each timestep of the model and therefore indicates how fast prior expectations (e.g., about likely ball location) are being adjusted in response to sensory observations (e.g., about the ball’s actual observed location). It is worth noting that, in this context, learning rate does not refer to improving at the experimental task; indeed, a high learning rate could be detrimental to task performance as it equates to disregarding potentially valuable prior observations (e.g., beliefs which have been formed through previous sensorimotor experiences and longer-term exposure to interception cue-outcome contingencies). Action precision refers to the mapping between beliefs and actions, it encodes the degree to which action choices are deterministic or random. Loss aversion describes the strength of the prior preference against observing a particular outcome.

Next we fit the models to observed data and extracted participant-wise estimated parameters for learning rate, action precision, and loss aversion. Average action probabilities (i.e., the probability of a participant’s actions under the model) were 73% suggesting high explanatory power of the model. Next, we checked parameter recoverability and independence (https://osf.io/2amdv/ – supplementary 4). Finally, the estimated model parameters were subject to between-condition comparisons (see *Results*).

### Procedure

Participants attended the lab for a single visit lasting ~ 60 min. They first completed an informed consent form and were fitted with the heart rate chest strap. While they were seated, their baseline heart rate was recorded over a period of 2 min. All participants then watched a short video explaining the broad aims of the study and what would be required during the experiment, to standardise the instructions. Next, participants were fitted with the VR headset and performed a familiarisation block of the interception task, consisting of four trials (two left and two right-sided trials). They then completed the high and low threat conditions in their preassigned (counterbalanced) order. Before each condition, participants watched a pre-recorded video (available from: https://osf.io/2amdv/) which explained the task to them. They then completed the S-ANX measure (anxiety thermometer) immediately after the video and before the interception task, capturing their anxiety about the upcoming event. The in-built eye trackers were calibrated at the start of the first block and on any obvious displacement of the headset. Participants then completed 36 interception trials in each of the high and low threat conditions. On each trial, participants began in the centre of the court, just behind the ‘short line’. The appearance of each ball was cued by three auditory tones which were followed by a variable onset delay. Based on previous pilot testing, the onset delay was randomly selected from a uniform distribution over a 0–5 s window. This variable onset window was required to create sufficient levels of challenge and uncertainty in the task. Participants then had to intercept the oncoming ball by reaching their racquet out to the left or right. When participants successfully intercepted the ball it ‘popped’, and a rewarding ‘ding’ sound was played, alongside a haptic vibration from the handheld controller. If the ball was missed, a ‘buzz’ sound was played.

### Anxiety induction

The anxiety induction consisted of a threat manipulation similar to that used in previous studies (Wood and Wilson 2010; Allsop and Gray 2014). This was based on the provision of evaluative and ego-threatening instructions, monetary incentives, and immediate consequences for performance failure. Two pre-recorded videos presented by the head of department were used to deliver these instructions to give them added legitimacy. During the *low-threat* block of trials, participants were advised that their score was not important and that they should just do their best. On the game screen, the score was replaced with ‘N/A’, and the text ‘not recording’ was shown in the bottom left (see Fig. 1). By contrast, in the *high-threat* block of trials, participants were told that they if they performed well enough they would receive a monetary prize, whereas if they scored in the bottom three, they would be consented into a follow-up study which examines people who choke under pressure. They were also told that at the end of testing a league table of participants' scores would be made public to all those who participated. On their head-set display, their score was displayed in front of them along with a sign saying ‘Recording’, to reinforce these elements of evaluative pressure.

### Data analysis

Processing of the raw eye movement data was performed in MATLAB 2022b (Mathworks, US) using bespoke analysis scripts which are available from the Open Science Framework project page (https://osf.io/2amdv/). Computational modelling was also performed using MATLAB, using scripts adapted from the Statistical Parametric Mapping 12 toolbox. Statistical analysis was performed in RStudio v1.4.1106 (R Core Team 2017). A series of linear mixed effects models (LMMs; fitted using restricted maximum likelihood in the lme4 package (Bates et al. 2014)) were used to examine the hypotheses. Model fit checks were performed using the ‘performance’ package (Lüdecke et al. 2021). For each model we report R^2^_conditional_ (overall) and R^2^_marginal_ (all fixed effects) effect sizes, as well as standardised beta effect sizes for each independent variable. Interpretations follow Acock's (2014) rule of thumb for std. beta values that < 0.2 is weak, 0.2–0.5 is moderate, and > 0.5 is strong.

## Results

### Manipulation checks

**Fig. 4:**
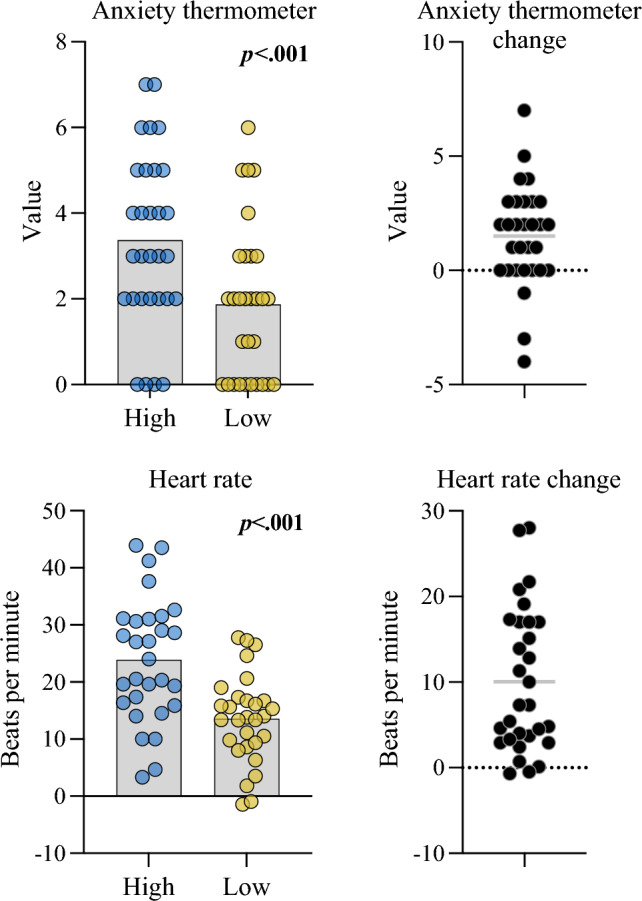
Anxiety and heart rate comparisons between low and high pressure conditions. Note: Plots show mean and overlaid data points for the condition comparison (left) and the associated between-condition difference (right; high minus low). Heart rate plots show HR (averaged across each condition) relative to baseline resting value

First we examined whether the pressure manipulation successfully increased state anxiety. A linear mixed model on S-ANX, with participant as a random effect [R^2^_conditional_ = 0.42; R^2^_marginal_ = 0.14] indicated that the effect of Condition [High] was statistically significant and large in magnitude [β = 1.50, 95%CI [0.69, 2.31], *p* < 0.001; std. beta = 0.73] indicating increased self-reported anxiety during the high pressure condition (see Fig. 4 top). The linear mixed model for HR [conditional R^2^ = 0.88; marginal R^2^ = 0.09] similarly indicated a statistically significant increase in the high pressure condition [β = 10.06, 95%CI [6.99, 13.12], *p* < 0.001; std. beta = 0.60] (see Fig. 4 bottom). Additionally, a further model indicated that there was a strong and statistically significant relationship between HR and S-ANX. The model, with participant as random effect, accounted for the majority of variance in S-ANX [R^2^_conditional_ = 0.76], of which a large amount was attributed to HR [R^2^_marginal_ = 0.22]. This indicated that changes in HR were strongly related to anxiety [β = 0.06, 95% CI [0.05, 0.06], *p* < 0.001; std. beta = 0.62], albeit with some remaining variance in S-ANX not explained by HR.

### Eye movement variables

#### Predictive eye movements

Before examining the model-derived variables, we first checked that there was no general difference in predictive gaze behaviours between high and low pressure conditions. We did not anticipate changes in these variables; however, we assessed them to determine whether any changes in model variables were simply a result of broad changes in the ability to make anticipatory eye movements. A linear mixed model on predictive gaze position (i.e., raw horizontal position) indicated that the effect of Condition [High] was statistically non-significant [β = 1.79e-03, 95% CI [-0.05, 0.05], *p* = 0.94; std. beta = 3.02e-03, R^2^_conditional_ = 0.14, R^2^_marginal_ < 0.01]. Similarly, a logistic model indicated that there was no change in the accuracy of pre-onset gaze predictions between conditions [β = -0.04, 95% CI [-0.21, 0.13], *p* = 0.68; std. beta = -0.04, R^2^_conditional_ < 0.01], i.e., participants did not attend to the correct side more/less frequently. We further examined whether there was any difference in variance of predictive gaze location, by computing the standard deviation in predictive gaze position for each participant. A linear model indicated that there was no effect of condition on the standard deviation of predictive gaze position [β = -0.02, 95% CI [-0.07, 0.03], *p* = 0.51; std. beta = -0.08, R^2^_conditional_ = 0.83, R^2^_marginal_ < 0.01]. These results indicate that there was no systematic difference in predictive gaze position, variance of that position, or the correctness of that position between high and low pressure conditions.

#### Active inference model variables

Next, we examined whether there were differences in active inference during anxiety, based on the model-estimated parameters. A linear mixed effects model indicated that there was no statistically significant effect of pressure condition on α (action precision) [β = 0.97, 95% CI [-0.23, 2.17], *p* = 0.11; std. beta = 0.33, R^2^_conditional_ = 0.40, R^2^_marginal_ = 0.03], showing no change in the randomness of action selection. Similarly, there was no effect of condition on the loss aversion parameter [β = -0.21, 95% CI [-0.85, 0.42], *p* = 0.51; std. beta = -0.08, R^2^_conditional_ = 0.80, R^2^_marginal_ = 0.002]. We did, however, observe a significant reduction in learning rate (η) in the high pressure condition [β = -0.09, 95% CI [-0.16, -0.02], *p* = 0.009; std. beta = -0.62, R^2^_conditional_ = 0.26, R^2^_marginal_ = 0.10], with a large effect indicating slower updating of gaze predictions under state anxiety.

We also examined whether there were relationships between the model-derived parameters and self-reported or physiological measures of anxiety. For change in self-reported anxiety (i.e., high pressure minus low pressure), the correlation with learning rate was weak and non-significant [*r* = -0.24, *p* = 0.21]. There were, however, significant relationships with α [*r* = -0.44, *p* = 0.02] and loss aversion [*r* = -0.51, *p* = 0.005] indicating that greater increases in felt anxiety were associated with more random and less deterministic action choices, as well as a reduced aversion for unpreferred outcomes. There were no correlations between change in HR and α [*r* = -0.12, *p* = 0.54], η [*r* = 0.04, *p* = 0.85], or loss aversion [*r* = -0.11, *p* = 0.57].

#### Pursuit gain

**Fig. 5:**
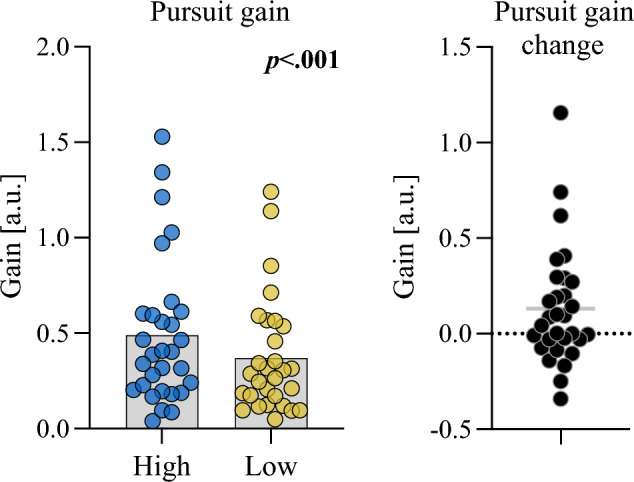
Comparison of pursuit gain between low and high pressure conditions. Note: Plots show mean and overlaid data points for the condition comparison (left) and the associated between-condition difference (right; high minus low)

To examine whether pressure condition also affected in-flight tracking, we ran a linear mixed model on log-transformed pursuit gain values. The model (with participant as random effect) had a conditional R^2^ of 0.40 and marginal R^2^ of 0.01. The effect of Condition [High] was statistically significant but small [β = 0.33, 95% CI [0.21, 0.45], *p* < 0.001; std. beta = 0.10] indicating higher gain values (closer to 1) under high pressure conditions (see Fig. 5 – top].

#### SD of pre-release position

**Fig. 6:**
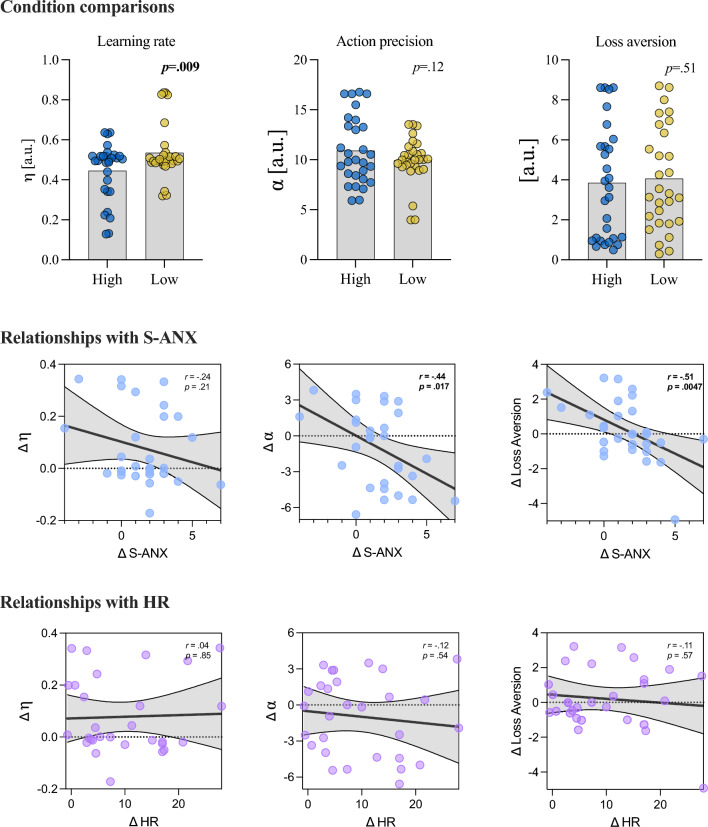
Active inference model comparisons between low and high pressure conditions and relationships with state anxiety. Note: Scatterplots show Pearson’s *r* values and (uncorrected) p-values

To examine whether pressure condition affected gaze sampling prior to ball release, we ran a linear mixed model on the two dimensional standard deviation of eye position for the period preceding the auditory cue. The model (with participant as random effect) had a conditional R^2^ of 0.41 and marginal R^2^ of 0.01. The effect of Condition [High] was statistically significant but relatively small [β = 0.04, 95% CI [0.02, 0.05], *p* < 0.001; std. beta = 0.15] indicating an increase in the variance of eye position before ball release under high pressure (Fig. 6).

#### Entropy

Finally, we tested whether the complexity of pre-release sampling was different between conditions. A linear mixed model on sample entropy of eye position indicated a small but statistically significant difference between the low and high pressure conditions for both the horizontal [β = 0.01, 95% CI [3.38e-03, 0.01], *p* = 0.002; std. beta = 0.12] and vertical [β = 0.01, 95% CI [3.38e-03, 0.01], *p* = 0.036; std. beta = 0.09] planes. This indicates a higher degree of randomness or complexity in eye movements in the high pressure condition.

## Discussion

In the present work, we examined how eye movements, and their underpinning predictive mechanisms (e.g., uncertainty-related learning and belief updating) were affected by temporary states of performance anxiety. Based on EMU and FEP accounts of anxiety (Hirsh et al. 2012; McGovern et al. 2022), we hypothesised that performance pressure would increase entropy internal to the agent and reduce participants’ ability to predict future states (impaired ball tracking), which they would seek to resolve by: i) constraining updates to predictive eye movements; and ii) more reflexive sampling of the task environment. Our hypotheses about resolving internal entropy through reduced learning rates and increased visual sampling were supported by the data. However, we found no disruption to in-flight tracking during the high-pressure condition. Overall, these findings, based on advanced modelling techniques, support the theoretical position that updating of prior beliefs may be impaired during instances of performance anxiety.

Firstly, the manipulation checks indicated that we successfully increased participants’ state anxiety, as shown by clear increases in S-ANX and HR in the high (compared to the low) pressure conditions. Our primary hypothesis was that *updating* of predictive eye movements (i.e., not actual pre-release position but trial-to-trial changes) would be impaired under state anxiety. Model-based estimates of trial-to-trial updating indeed indicated a lower learning rate in the high pressure (anxiety) condition. This finding accords with previous work reporting disrupted reward processing and updating of motor predictions under anxiety (Sporn et al. 2020). Adding to this picture, Lang et al. (2015) found more rigid, repetitive, and redundant hand movement patterns during state anxiety, which they characterised as an attempt to return to familiar low-entropy states. Taken together, these results suggest a growing picture that people attempt to counter uncertainty (either in the form of internal entropy or shifting environmental probabilities) by moving towards more familiar and certain sensorimotor patterns.

The slowed updating of sensorimotor predictions observed here contrasts with the relationship between anxiety and *increased* rates of learning (belief updating) reported by Lawson et al. (2021). However, this apparent discrepancy is easily resolved by appealing to active inference models of perception and action (Friston et al. 2009, 2010). Within active inference theory, a distinction is made between minimising two types of ‘prediction error’ known as *variational free energy* (VFE) and *expected free energy* (EFE). VFE is effectively momentary prediction error arising from inferences about the likely causes of sensations (e.g., during simple perceptual or associative learning tasks). By contrast, EFE refers to minimisation of *future* prediction errors through action. The ability to infer future states (of the body or world) with confidence is critical in computing EFE, as action policies are most likely to be selected when they (i) minimize uncertainty over states and (ii) maximize the probability of preferred observations. Uncertainty about future states (i.e., expected precision) is explicitly represented by the *E* term in our active inference model in Fig. 3. Consequently, if an organism is generally less confident predicting its own future states, it is unlikely to switch to new actions where the outcomes may be more uncertain. In this way, perceptual beliefs about the world (e.g., during associative learning tasks; Lawson et al. 2021) may be updated more quickly during states of uncertainty (Behrens et al. 2007), while beliefs driving actions (e.g., predictive eye movements in our task) may not. A question for future work is, therefore, whether learning rates for beliefs about environmental states can diverge from those relating to sensorimotor predictions during the same task.

There are additional variables to consider that may influence this relationship between uncertainty and belief updating. For instance, ‘degrees of uncertainty’ are hard to compare between studies/tasks, therefore results from associative learning and motor learning studies may not be directly comparable. Prediction updating is also dependent on higher-level beliefs about uncertainty (Mathys et al. 2014; Heilbron and Meyniel 2019). Indeed, probabilistic relationships in the world can themselves be more or less changeable (Bland and Schaefer 2012), and this additional ‘volatility’ can, itself, have varying levels of stability. When uncertainty is short-lived, faster prediction updating will be adaptive, but if it is persistent, updating your predictions is ineffective and wasteful. The source of uncertainty is, therefore, also relevant. For instance, if uncertainty is purely a function of probabilistic relationships in the environment and the outcome of one’s actions can be estimated with certainty, predictive movements should be adapted more quickly. If, on the other hand, the organism has high internal entropy (e.g., anxiety) and cannot predict their own future states with assurance, prediction updating may be slower. Given the widespread impact that these variables can have on performance and learning, future investigations may wish to explore the (inter-) relationships between uncertainty, volatility, and anxiety beliefs during naturalistic sensorimotor tasks.

We also observed relationships between S-ANX and both loss aversion and action precision parameters from the active inference model. There was a weak non-significant relationship, in the expected direction, for learning rate. As the analyses of loss aversion and action precision were exploratory, they should, however, be interpreted with more caution. The relationship between S-ANX and reduced action precision (i.e., less deterministic mapping from beliefs to choices) makes intuitive sense and aligns with the theoretical models we have discussed above. Indeed, states of high entropy can mean that an organism is less able to predict its current or future states with confidence, so there may be an increase in the apparent randomness of their sensorimotor actions (Friston 2010; Hirsh et al. 2012). More broadly, anxiety is characterised by heightened uncertainty and a tendency to prioritise safety or avoidance behaviours over precise, goal-directed actions. Reduced action precision can, therefore, be interpreted as a diminished confidence in selecting specific actions, potentially reflecting the individual's increased sensitivity to uncertainty or fear of negative outcomes. This finding is relevant to real-world decision-making during high-anxiety states, where individuals might exhibit hesitation, reliance on habitual actions, or less optimal choices in uncertain environments (Browning et al. 2015; Huang et al. 2017; Hein et al. 2021). The observed relationship between increasing S-ANX and reduced loss aversion, however, seems in the opposite direction to what we would generally expect for anxious emotional states. Generally, research has suggested a positive association between these constructs, particularly in perceptual decision-making tasks (Hartley and Phelps 2012). Sporn et al. (2020) have previously reported that anxiety disrupts reward processing in a motor learning task, so this unexpected result could be related to a reduced ability to predict the rewards associated their own actions (eye movements).

Turning to our secondary hypotheses, we found that in-flight gaze tracking was not disrupted under performance anxiety. In fact, participants in the high-pressure condition actually had higher pursuit-gain values, indicating that they were following the path of the ball more closely and continuously than in the low-pressure conditions. This effect was unexpected but might be an artefact of an increased motivation to succeed in the high pressure conditions, given the performance incentives. Our hypotheses related to increased sampling of the environment prior to release were, however, supported. We observed small but statistically significant increases in both the variability and the complexity of gaze position before target release. These findings align with previous studies that have reported similar anxiety-induced disruptions to gaze control (Janelle 2002; Wilson et al. 2009; Allsop and Gray 2014; Vine et al. 2015). Many of these studies have proposed attentional mechanisms (Payne et al. 2018) – such as an altered balance between top-down and bottom-up systems (Corbetta and Shulman 2002) – as responsible for these effects. We have instead suggested that they could be conceptualised as an active attempt to reduce uncertainty in the environment through increased sampling of visual information (e.g., as in Arthur et al. 2020). While attentional mechanisms could equally explain the changes to gaze variability and entropy, the alterations to learning rate that we observed are more consistent with an entropy reduction explanation. Additionally, the observed increase in pursuit gain during the high pressure condition is inconsistent with an attentional explanation but could be interpreted as an additional attempt at uncertainty reduction through closer ball tracking.

The present work has both theoretical and practical implications. Our results contribute to an improved theoretical understanding of the interactions between motor and affective states, illustrating that the feedforward and predictive mechanisms which are fundamental to sensorimotor control may be impaired during high performance pressure (or high anxiety) conditions. Our findings align with EMU and FEP accounts of anxiety (Hirsh et al. 2012; McGovern et al. 2022). The results might also have implications for our understanding of motor skill breakdowns under pressure, such as during sports. While previous explanations have focused on attentional explanations (Payne et al. 2018), our findings suggest that anxiety might disrupt predictive mechanisms, which could theoretically contribute to episodes of ‘choking’ (see Harris et al. 2023b for a review). Finally, our results have implications for designing motor skill training programmes. It has previously been suggested that training in the presence of anxiety may ‘inoculate’ sports performers to the subsequent effects of performance pressure (Low et al. 2021). Our results, however, suggest that the presence of anxiety during training could alter the learning of critical predictive sensorimotor control mechanisms. As such, it is crucial to understand these effects more deeply in order to determine the likely value of such ‘stress inoculation training’.

### Limitations

A limitation of the current work is that we most probably did not achieve genuinely high states of anxiety, such as would occur during public speaking, job interviews, or high-pressure sporting events. Scores on the anxiety thermometer unmistakably increased in the high pressure condition (with a large effect size) yet were still in the mid-to-low range of the self-report scale. For instance, no participants reported scores of eight or nine out of ten. Hence, the conditions in the experiment cannot be said to replicate some of these real-world experiences of anxiety. We did, however, observe large increases in HR suggesting a notable activation of the sympathetic nervous system. A related challenge is discerning whether the observed effects were driven by changes in cognitive anxiety or simple increases in arousal. The correlations between changes in S-ANX and HR and the model-derived variables indicated that relationships were much stronger for S-ANX than HR, suggesting that psychological anxiety, rather than just physiological arousal, was responsible. Future studies, however, should examine this issue further using self-report measures that distinguish between cognitive and somatic elements of anxiety (e.g., see Thomas et al. 2002).

The focus of the present work was on the mechanistic questions around updating of priors and subtle changes in eye movements. We cannot, currently, draw any conclusions about whether the observed changes will relate to overall changes in task performance. Indeed, changes in gross outcomes are likely to be subtle and challenging to detect. The only way to assess performance in this task was whether participants successfully intercepted the ball or not, whereas previous studies of anxiety typically measure outcomes like accuracy or performance variability. So future work could use a task that allowed these more sensitive changes in task execution to be studied and related to changes in model parameters.

In future work, it would be valuable to directly compare active inference to alternative reinforcement learning approaches, such as those adopting control as inference (CAI; Tschantz et al. 2020; Millidge et al. 2020)). While both frameworks share foundational principles, their treatment of ‘reward’ and decision-making diverges, with CAI optimizing external reward functions and active inference embedding preferences within the generative model (Friston et al. 2009). A comparative analysis would help delineate their respective strengths in capturing exploratory versus goal-directed behaviours, further refining their application to domains like performance anxiety and adaptive decision-making.

## Conclusion

They key finding from this work was that high pressure conditions can disrupt the updating of beliefs that underpin predictive sensorimotor control. In addition, we found performance anxiety to be related to a less deterministic mapping of beliefs to actions. These findings have relevance for a better understanding of how finely tuned motor skills can break down in the presence of heightened emotional states, as well as providing an empirical test of theories like the EMU and free energy principle (Hirsh et al. 2012; McGovern et al. 2022). By conceptualising anxiety as a state of uncertainty, and anxious behaviours as uncertainty reduction attempts, it will be possible to situate our understanding within a wider computational framework (Hirsh et al. 2012; Cornwell et al. 2017; McGovern et al. 2022) and potentially explain wide-ranging anxiety-related behaviours. This may contribute to the growing appreciation of uncertainty computations in both diagnosis (Carleton et al. 2012) and treatment (Boswell et al. 2013) of clinical anxiety, as well as situation-specific patterns of sensory processing and/or motor performance.

## Data Availability

All relevant data and code is available online from: https://osf.io/2amdv/

## Footnotes

1 Here we refer to aspects of movement that are driven by expectations, like anticipatory saccades that jump ahead of a moving target or looking towards the most likely ball release location, rather than predictive mechanisms like efference copy.
